# Regulation of Inflammation Pathways and Inflammasome by Sex Steroid Hormones in Endometriosis

**DOI:** 10.3389/fendo.2019.00935

**Published:** 2020-01-29

**Authors:** Elizabeth García-Gómez, Edgar Ricardo Vázquez-Martínez, Christian Reyes-Mayoral, Oliver Paul Cruz-Orozco, Ignacio Camacho-Arroyo, Marco Cerbón

**Affiliations:** ^1^Unidad de Investigación en Reproducción Humana, Consejo Nacional de Ciencia y Tecnología (CONACyT)-Instituto Nacional de Perinatología, Mexico City, Mexico; ^2^Unidad de Investigación en Reproducción Humana, Instituto Nacional de Perinatología-Facultad de Química, Universidad Nacional Autónoma de México, Mexico City, Mexico; ^3^Departamento de Ginecología, Instituto Nacional de Perinatología, Mexico City, Mexico

**Keywords:** endometriosis, inflammation, pro-inflammatory factors, inflammasome, sex steroid hormones, progesterone receptor, estrogen receptor, bacteria

## Abstract

Endometriosis is a gynecological disorder characterized by the growth of endometrial tissue (glands and stroma) outside the uterus, mainly in the peritoneal cavity, ovaries, and intestines. This condition shows estrogen dependency and progesterone resistance, and it has been associated with chronic inflammation, severe pain, and infertility, which negatively affect the quality of life in reproductive women. The molecular mechanisms involved in the pathogenesis of endometriosis are not completely understood; however, inflammation plays a key role in the pathophysiology of the disease, mainly by altering the function of immune cells (macrophages, natural killer, and T cells) and increasing levels of pro-inflammatory mediators in the peritoneal cavity, endometrium, and blood. These immune alterations inhibit apoptotic pathways and promote adhesion and proliferation of endometriotic cells, as well as angiogenesis and neurogenesis in endometriotic lesions. It has been demonstrated that hormonal alterations in endometriosis are related to the inflammatory unbalance in this disease. Particularly, steroid hormones (mainly estradiol) promote the expression and release of pro-inflammatory factors. Excessive inflammation in endometriosis contributes to changes of hormonal regulation by modulating sex steroid receptors expression and increasing aromatase activity. In addition, dysregulation of the inflammasome pathway, mediated by an alteration of cellular responses to steroid hormones, participates in disease progression through preventing cell death, promoting adhesion, invasion, and cell proliferation. Furthermore, inflammation is involved in endometriosis-associated infertility, which alters endometrium receptivity by impairing biochemical responses and decidualization. The purpose of this review is to present current research about the role of inflammasome in the pathogenesis of endometriosis as well as the molecular role of sex hormones in the inflammatory responses in endometriosis.

## Introduction

Endometriosis is a multifaceted gynecological condition with an estimated prevalence of ~10–15% of the general population (1). It is histologically defined as the presence of glands and stroma of endometrial tissue outside the uterus, mainly in the peritoneal cavity and ovaries (2–4). Reports have shown the location of these lesions in sub-peritoneal fat, recto-vaginal or recto-uterine spaces, bowel, bladder, pelvic nerves, uterosacral ligaments, ureters, anterior abdominal wall, as well as abdominal skin, diaphragm, pleura, lungs, pericardium, and brain, although these locations are usually less frequent than in pelvic structures (4–6).

Endometriotic lesions include superficial lesions in peritoneum and serosa, up to ovarian cysts (endometriomas), deep nodules, and severe adhesions (7). Pelvic endometriotic lesions have been systematically classified into superficial implants (peritoneum and ovarian cysts) and deep nodules (parametrium, Douglas pouch, the anterior wall of the rectum, vaginal wall, vesicouterine space, detrusor muscle of the bladder, ureters, and sigmoid colon) (5). The most widely used staging system of endometriosis was defined by the American Society for Reproductive Medicine (ASRM) in 1997, which classifies endometriosis severity into four different stages (I–IV), from minimal to severe, according to the score obtained from the size of the endometrial implants, involved pelvic structures, and spread of the lesions (7).

Endometriosis is the more frequent cause of chronic and cyclic pelvic pain in reproductive-age women, even occurring in adolescents and menopausal women (7); endometriosis encompasses different pain classes, including dysmenorrhea, dyspareunia, dysuria, and dyschezia (8, 9). Especially in cases of deep endometriosis, the pain is due to an invasion of endometrial cells and pro-inflammatory mediators on the nerve fibers, which trigger a disorder of nociceptive modulation of pain increasing the intensity of the neuronal signal toward the somatosensory cerebral cortex (10). Infertility is another consequence of endometriosis, by reducing implantation capacity, increasing risk of pregnancy loss and physical obstruction imposed by endometriotic lesions (11). Moreover, endometriosis symptoms negatively influence women's life quality by affecting their productivity, social life, and emotional health (12).

Treatment for endometriosis usually includes hormonal management and surgical elimination of lesions (10). Hormonal treatment consists of suppression of growth lesions and pain reduction by abolishing ovulation and menstruation through the administration of progestins, oral contraceptives, and gonadotropin-releasing hormone agonists (13). Surgical elimination is frequently made by laparoscopy, the gold standard for diagnosis and treatment, by which peritoneal implants, deep nodules, and ovarian cysts are removed; moreover, this technique is also performed for more radical proceedings as hysterectomy (1, 14). However, there is not an actual cure for the disease, and lesions and pain tend to reappear after treatments (15); therefore, it is important to continue the development of cutting-edge research focused on studying the underlying mechanisms involved in endometriosis pathophysiology.

There are several and not fully confirmed theories that describe endometriosis pathogenesis. The more accepted theory is the origin of lesions from retrograde menstruation, which establishes that during menstruation, residual endometrial tissue reaches the pelvic cavity, by traveling through fallopian tubes, due to uterine contraction disorders (3). This phenomenon is observed in 90% of women in reproductive age; however, it does not explain why only 10% of them develop endometriosis or the presence of lesions in more distal locations (14). Among other proposed theories are the coelomic metaplasia and the theory of Müllerian remnant; the first one involves the transformation of healthy peritoneal tissue into ectopic endometrial tissue; this theory is based on the fact that peritoneal and endometrial cells have a common origin from coelomic epithelium. In contrast, vascular and lymphatic metastasis suggests that reminiscent endometrial tissue travel through the blood and lymphatic vessels to reach ectopic locations; on the other hand, the theory of Müllerian remnant argues that cellular debris from embryonic Müllerian duct transform into endometriotic tissue by the influence of sex hormones rising at the beginning of puberty (3). There are efforts to unify the existent theories (16); however, the precise mechanisms underlying origin and development of endometriosis remain mainly unknown.

At a cellular level, the main alterations in endometriosis are characterized by cell proliferation, inflammation, and angiogenesis, which are closely connected to each other and are caused by an alteration in sex hormonal signaling, that depend on the sustained activation of estradiol (E2)-dependent pathways and the disruption of those dependent on progesterone (P4), through alteration of activity of their cognate receptors. This alteration in the activity of hormone receptors converges in a distinctive phenotype of resistance to progesterone and of estrogen dependence. A recent and detailed revision about the role of P4 and E2 in endometriosis describes the normal molecular hormonal regulation of the physiology of endometrium and its alterations in endometriosis (17) and highlights inflammation as a known key contributor in the pathophysiology of endometriosis, which is strongly associated with chronic pelvic pain and defects in endometrial receptivity and the decidualization process (17, 18). In fact, endometriosis is frequently considered as an inflammatory disease. Therefore, this review presents the current research about the role of inflammation and the inflammasome in the pathogenesis of endometriosis, as well as a discussion about the molecular mechanisms involved in the alteration of immune and inflammatory responses by female steroid hormones in this disease.

## Pathogenesis of Endometriosis: Altered Pathways

Hormonal imbalance in endometriosis is the main contributor in the alterations of multiple cellular functions such as proliferation, adhesion, and differentiation, as well as evasion of immune clearance, neurogenesis, angiogenesis, pain generation, metabolism, and inflammation (3). This imbalance is due to an increased expression of aromatase (CYP19A1) (19) and a decreased expression of progesterone-regulated 17β-hydroxysteroid dehydrogenase (17β-HSD) (20), which increases bioavailable levels of E2 in ovaries, peripheral tissue, and endometriotic lesions (21). Besides enzymatic and hormonal changes, the activity of nuclear receptors is also modified, through dysregulation of their expression at both mRNA and protein levels (22, 23). In the case of estrogen receptors (ER), there is an increase of isotype ER-β (*ESR2*) expression associated with hypomethylation of its promoter (24), and a significant reduction of ER-α (*ESR1*) expression by hypermethylation of its promoter and through direct inhibition by ER-β. In contrast, both progesterone receptor isoforms PR-A and PR-B (*PRG*) show a decreased expression; in particular, PR-B undergoes a drastic downregulation (mRNA and protein levels), which is mainly due to hypermethylation of the PR-B promoter (25), therefore affecting downstream hormone target genes (26).

The cellular and molecular processes involved in the development of endometriosis are only partially described. Considering retrograde menstruation as the potential beginning of endometriosis, it has been proposed that viable glandular and stromal endometrial cells contained in the menstrual debris reach the peritoneal cavity and are able to adhere to the peritoneum by interaction of cell surface receptors such as integrins, with membrane adhesion molecules of extracellular matrix, like fibronectin and laminin (27, 28). Besides, this debris contains stem cells that could also be responsible for the progress of endometriosis, by differentiating in endometrial cells that are unable to decidualize and that possess a phenotype of progesterone resistance and immune function alteration (29).

Furthermore, it has been shown that after initial adhesion, endometriotic cells invade peritoneum, possibly through the action of matrix metalloproteinases (MMPs) that degrade the basal lamina containing laminin, fibronectin, and collagen, which allows the remodeling of surrounding tissue (30). Additionally, reports have demonstrated that E2, through the increased activity of ER-β, promotes the cellular survival, by impairing tumor necrosis factor-α (TNF-α)-mediated apoptosis (31), increasing cellular proliferation with the participation of growth factor signaling, and favoring epithelial–mesenchymal transition that contributes to the production of collagen and formation of fibrosis (7). Also, ER-β induces the proliferation and survival of endometriotic cells through the increase of mRNA and protein levels of Ras-like estrogen-regulated growth inhibitor (RERG) that induces ribosomal biogenesis, while significantly stimulating expression of glucocorticoid-regulated kinase (SGK1) that has an anti-apoptotic role (32, 33). Growth factors such as hepatocyte growth factor (HGF), epidermal growth factor (EGF), platelet-derived growth factor (PDGF), insulin-like growth factors (IGF), and basic fibroblast growth factor (BFGF) also participate in the progression of endometriotic lesion through their strong mitogenic activity (3).

Additionally, studies have shown that an immunologically permissive environment in the peritoneum (34) is involved in endometriosis pathogenesis. Impaired inflammatory cell function of macrophages with a reduced phagocytic activity (35) and a resistance of endometriotic tissue to be lysed by natural killer cells (NKs) (36) allow the evasion of clearance by the immune system.

Once invasion takes place, angiogenesis and neurogenesis are activated coordinately. Angiogenesis allows the maintenance of lesions, supplying them with functional blood vessels that form a dense vascularization. In this process, diverse growth and pro-angiogenic factors play important roles, such as vascular endothelial growth factor (VEGF) that is regulated by E2 and responds to an inflammatory microenvironment, promoting endothelial cell proliferation (37, 38). Neurogenesis is linked to both inflammatory response and angiogenesis and, along with an imbalance in sensory and sympathetic innervation, contributes to the growth of nerve fibers, subsequent peripheral neuroinflammation, and generation of chronic pain (39). One of the postulated consequences of immune cell activation in the endometriosis microenvironment is the production of cytokines, growth factors, and eicosanoids that simultaneously stimulate lesion innervation and neovascularization through a coordinated mechanism that is known as neuroangiogenesis (40).

Concerning the molecular origin of these alterations, there is an increasing evidence of genetic and epigenetic changes in epithelial and stromal cells, respectively, both in ectopic and eutopic endometrial tissue compared to healthy endometrium. This changes influence of all aspects in the pathophysiology of endometriosis, by modifying the expression of essential components of cellular and biochemical pathways compromised in endometriosis, including the expression of ER and PR genes, and can explain associated inheritance and predisposition to present the disease (41).

Some genetic variants associated with endometriosis risk have been linked to chromosomic regions 7p15.2 and 10q26 by genetic linkage studies. These regions contain *CYP2C19* (cytochrome P450 2C19), *INHBA* (inhibin subunit beta A), *SFRP4* (secreted frizzled-related protein 4), and *HOXA10* (homeobox A10) genes (42). On the other hand, genome-wide association studies have shown 14 genetic loci associated with endometriosis, which are involved in alterations of wingless-related integration site protein (WNT), mitogen-activated protein kinase (MAPK), and signal transducer and activator of transcription 3 (STAT3) signaling (7). Remarkably, cancer driver mutations have been identified in *ARID1A, PIK3CA, KRAS*, and *PPP2R1A* genes in epithelial cells of endometriotic tissue; however, it has not yet been demonstrated that these changes originate malignant transformation from endometriotic lesions (43).

Different transcriptomic alterations have been detected in endometriosis patients; for example, by using cDNA microarray analysis specific genes that mainly encode components of the immune system and inflammatory pathways, proteins involved in cell adhesion and remodeling of the extracellular matrix as well components of signal transduction pathways were found differentially expressed in ectopic endometrium when compared to eutopic endometrium; some altered genes are those that encode phospholipase A2 group IIA (PLA2 IIA), PLA2 group V (PLA2 V), fatty acid–binding protein 4 (FABP4), prostacyclin synthase (PGIS), complement component 7, claudin 11, heptoglobin, some integrins, and tissue inhibitors of metalloproteinases 1 and 2 (TIMP-1 and TIMP-2) (44). Furthermore, next-generation sequencing analysis of eutopic endometrium transcriptome has shown abnormalities in comparison with endometrium from healthy women, demonstrating differential expression of genes involved in extracellular matrix remodeling, angiogenesis, cell proliferation and differentiation, such as matrix metallopeptidase 11 (MMP-11), dual specific phosphatase 1 (DUSP1), Fos proto-oncogene (FOS), serpin family E member 1 (SERPINE1), and adenosine deaminase 2 (ADA2) (45).

The regulation of gene expression by epigenetic mechanisms encompasses DNA methylation, post-translational modifications of histones, non-coding RNAs (mainly microRNAs), among others (46). The role of epigenetic mechanisms in the pathogenesis of endometriosis has been recently explored and reviewed (47). Genome-wide DNA methylation studies have shown that endometriotic lesions and eutopic endometrium display an altered epigenetic program compared with endometrial tissue from women without the disease, which in turn has been associated with an altered expression profile in several genes involved in the pathogenesis of endometriosis (29, 47–49). Particularly, an increase in the content of DNA methylation has been reported in the promoter and coding region of *GATA2* gene, and the promoter of *ESR1* and *PGR* genes in endometriotic cells in relation to endometrial cells, whereas *GATA6, ESR2*, and *SF1* genes are hypomethylated in endometriotic cells (24, 25, 50, 51). These alterations were associated with the corresponding changes in gene expression, which partly explains the altered progesterone signaling, progesterone resistance, increased inflammation, and the excessive estradiol production observed in this disease (47). Moreover, it has been suggested that histone acetylation and methylation are also involved in the pathogenesis of endometriosis, since alterations in those post-translational modifications have been associated with the presence of the disease (52).

In spite of being considered a “benign” disease, the complexity of endometriosis is very clear. Its pathogenesis is associated with different molecular and cellular alterations in endometriotic tissue and the surrounding microenvironment; these modifications are closely related to each other and form a complex positive feedback loop, which indicates that probably there is not only one mechanism that originates and influences their pathogenesis. Components of the molecular mechanisms involved in endometriosis pathophysiology show high heterogeneity between patients, notwithstanding that they are analyzed in populations as homogeneous as possible. Indeed, great recent advances in the knowledge about the disease have been made; however, there is still a gap in the information that allows the identification of the key pathway or pathways that start the appearance of endometriotic lesions. According to recent findings, we consider that the phenomenon where all mechanisms converged is the chronic inflammation, which is present in all the clinical manifestations of this gynecological disorder, without forgetting that it is subject to a fine hormonal regulation. For that reason, in the next sections, we described main cellular and molecular alterations of the immune response in endometriosis.

## Alteration of Inflammatory Functions in Endometriosis

Inflammation is the main molecular and cellular process involved in the pathophysiology of endometriosis, causing pain, tissue remodeling, lesion formation, fibrosis, and infertility (6). Aberrant production and secretion of immune mediators like cytokines, prostaglandins, and metalloproteinases, as well as alteration of activity and infiltration of immune cells in sites of lesion and peritoneal cavity are some changes involved in this disrupted response (3, 53).

**Figure 1:**
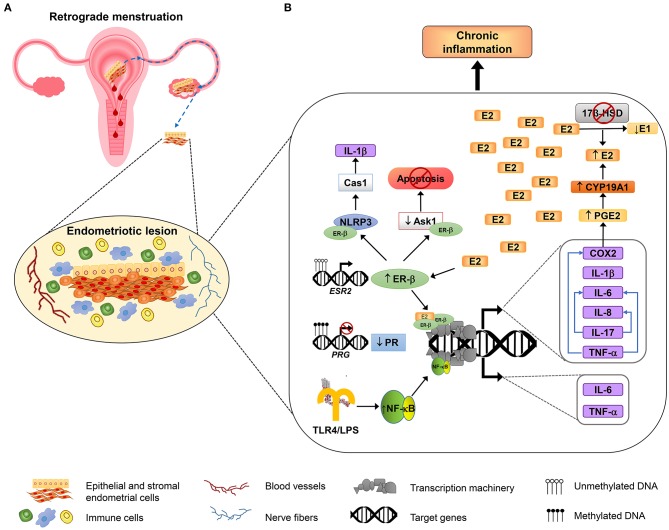
Inflammatory mechanisms involved in endometriosis. An overview about the molecular mechanisms involved in the adhesion, survival, proliferation, inflammation, and neuroangiogenesis of endometriotic lesions. **(A)** According to the most accepted hypothesis of endometriosis origin, stromal and epithelial cells from shed endometrial tissue reach ovaries or peritoneal cavity by retrograde efflux during menstruation. The developing endometriotic lesion stimulates angiogenesis and nerve development and secretes chemoattractant molecules recruiting high numbers of macrophages (showed in blue) and natural killer cells (indicated in green) with reduced phagocytic and cytolytic activity, respectively. **(B)** Alterations in DNA methylation lead to the growth and maintenance of endometriotic lesions by increasing the expression of estrogen receptor β (ER-β) and reducing progesterone receptor (PR) expression. Overexpressed ER-β induces the expression of genes that encode several pro-inflammatory molecules, such as IL-1β, IL-6, IL-8, IL-17, TNF-α, and COX2; some of them, in turn, can stimulate the production of other immune molecules (depicted as blue arrows). COX2 promotes an increase of the synthesis of prostaglandin E2 (PGE_2_), and PGE_2_ induces aromatase activity (CYP19A1), leading to E2 increased levels. E2 elevated levels are sustained by reduced expression of 17β-HSD, an enzyme that catalyzes the conversion of E2 to estrone (E1), and that is induced by PR (not shown). In addition, ER-β interacts with some inflammasome components such as NLRP3 sensor and caspase 1 (Cas1) to activate IL-1β, and also interacts with apoptosis signal-regulating kinase-1 (ASK-1) to reduce its activity and hence inhibits TNF-α-mediated apoptosis. On the other hand, the induction of genes involved in the inflammatory response in endometriosis is also mediated by NF-κB activation, through the TLR4 interaction with *E. coli* LPS, or possibly through other microbial metabolites associated with infections or dysbiosis, as is proposed by “bacterial contamination” theory.

The interaction between immune and hormonal systems considerably impacts endometriosis pathogenesis and development (14). Hormonal signaling differentially regulates immune response; P4 is known by its anti-inflammatory capacity, mediated mainly through PR-B, which overcomes nuclear factor kappa-light-chain-enhancer of active B cells (NF-κB) signaling (54). On the other hand, E2 has a noteworthy role in the promotion of inflammation, by inducing the secretion of cytokines and prostaglandins from peritoneal macrophages (55) by action of ER-β (23), while ER-α has a dual role, with both anti- and pro-inflammatory effects (56). However, in endometriosis, there is an imbalance in the functions of sex steroid hormones, with an important role of estrogens in the exacerbated pro-inflammatory state of endometriosis. Changes induced by sex steroid hormones through their cognate receptors at the immune and inflammatory levels are described below. Figure 1 shows a schematic model that resumes the mechanisms involved in regulation of inflammation in endometriosis.

### Alteration of Immune Cell Function

Inflammation in endometriosis is generally attributed to the recruitment of macrophages and other activated leukocytes from bone marrow to the developing endometriotic lesions and eutopic endometrium, these cells are attracted by chemokines synthesized and released *in situ*. In the endometriotic lesions, immune cells secrete elevated levels of pro-inflammatory cytokines, which in turn stimulate the production of diverse molecules such as chemokines, and growth factors that sustain an inflammatory microenvironment and the remodeling of the ectopic tissue (40). This tissue also has the capacity of releasing pro-inflammatory factors, creating a positive feedback loop that maintains the chronic inflammation state (57).

Macrophages are the first defense line of the immune system, by removing pathogens through phagocytosis and cell debris through cytokine secretion (58). An elevated number of macrophages are found in endometriosis patients during all phases of the menstrual cycle (59). Interleukin-8 (IL-8), C-C chemokine regulated on activation normal T cell expressed and secreted (RANTES, or CCL5), as well as monocyte chemotactic protein-1 (MCP-1, or CCL2) have been found in peritoneal fluid mainly functioning as chemoattractants of recruited macrophages (60–63). As a result, peritoneal macrophages from endometriosis patients overexpress cyclo-oxygenase 2 (COX-2) and secrete higher levels of prostaglandins than those from women without disease (64).

MMPs normally regulate macrophage activity by degrading the extracellular matrix of cells that will be phagocyted, but these molecules show a reduced expression in macrophages of patients with endometriosis, which is subsequently associated with impaired phagocytic activity. Particularly, MMP-9 has a reduction in both expression (mRNA and protein) and enzymatic activity, and this reduction is essentially due to the presence of prostaglandin E_2_ (PGE_2_) in the peritoneal fluid. Moreover, PGE_2_ activity takes place through the EP2/EP4 (receptors for MMPs)-dependent PKA pathway (65). Other studies report that phagocytosis inactivation by PGE2 trough CD36 inhibition is mediated by the EP-2. On the other hand, cytokines secretion activity by these cells is increased; for example, IL-8, IL-10, and tumor necrosis factor (TNF)-α are found in peritoneal fluid of endometriosis patients at greater levels than fertile women (66); thus, macrophages could promote and sustain an inflammatory environment required for endometriosis progress (67).

Lymphocytes are essential to determining survival, implantation, and proliferation of endometriotic cells (68); among affected lymphocytes in endometriosis are T-cells. Usually, there is a balance between populations of regulatory T-cells (T_regs_), a subgroup of helper T cells (CD4+) that act as anti-inflammatory cells, and effector or cytotoxic T-cells (CD8+); this equilibrium is required to maintain immune tolerance and to eliminate endometriotic cells. However, the high levels of E2 and the reduced P4 response influence in the elevated concentration of T_regs_ in peritoneal fluid and in the endometriotic lesions, which decrease immune surveillance (58) and suppress the immune response, promote in this way the establishment of lesions (69). In contrast, effector T cells show a decreased activity, while helper T-cells in general are increased and participate in secreting high levels of cytokines (58, 70). Growth of endometriotic lesions is allowed by reduced ratios of Th1 to Th2 cells, with the consequent potentiation of Th1 response, mainly in the first stages of disease (71, 72). The Th1/Th2 ratio depends on the stage of the disease once it has been established, since Th1 cytokines prevalence occurs during minimal to mild endometriosis whereas Th2 cytokines are manifested in severe stages (58). Besides, the Th17 cell subset has a role in endometriosis, mediated by secretion of IL-17 resulting in the secretion of chemokine (C-C motif) ligand 20 (CCL20; also known as macrophage inflammatory protein-3, MIP3A) by endometriotic cells. CCL20 acts as a chemoattractant for Th17 cells and neutrophils to the endometriotic lesion (68). IL-17A also can stimulate IL-8 secretion and COX2 expression that promotes the proliferation of human endometriotic stromal cells (73).

NK cells represent around 15% of leukocytes in peripheral blood and of 30% of peritoneal leukocytes; the main function of NK cells consists in protecting against infections and tumor development through their cytolytic and immunomodulatory capabilities. NK cells destroy other cells by secreting lytic granules that contain granzymes, perforin, cytotoxins, or cytokines (74). NK cells in peritoneal fluid and peripheral blood from patients with diagnosed endometriosis have a decreased cytotoxicity against endometrial cells (75). Hence, they fail in eliminating endometrial fragments from menstrual debris in ectopic sites due to both their diminished activity and resistance from ectopic endometrium to be eliminated, which could be due to the presence of soluble non-specific factors released by human endometrial cells able to interfere with NK cell function, as suggested by the reduced activity of NKs in the presence of conditioned media obtained from human endometrial cell culture, without compromised cell viability (36). Alteration of NK cytotoxic activity in endometriosis patients is related to the reduced levels of granzyme B and perforin. In addition to defects in their activity, NK cells showed reduced numbers both in peritoneal fluid and in peripheral blood of endometriosis patients vs. control women (76, 77). Analysis of populations of peritoneal NK cells has shown that mature NK cells (CD32CD56+) are significantly decreased, in contrast with the proportions of immature NK cells (CD272CD11b2) that are increased, which suggest that the pathogenesis of endometriosis is associated with anomalous differentiation of NK cells (36). On the other hand, the reduction in NK activity correlates with the severity of disease (78), which makes them a potential marker and diagnosis tool of endometriosis and its severity. On the other hand, the reduction of NK function is associated with elevated levels of E2 in endometriosis. The association between NK cell activity and serum E2 levels suggests that immunoendocrine interaction is essential for the progression of endometriosis (77).

Mast cells are another group of leukocytes that are involved in endometriosis by modulating the recruitment, survival, development, phenotype, or function of other immune cells involved in endometriosis pathology, including macrophages, granulocytes, dendritic cells, and T and B cells (79). High numbers of degranulated mast cells have been found in endometriotic lesions, mainly of deep infiltrating type, but not in non-affected areas from peritoneum or eutopic endometrium of patients, indicating that a reaction of hypersensitivity is strongly associated to endometriosis (80); released mediators include TNF-α, IL-8, MCP-1, RANTES, and stem cell factor (SCF) (81). Additionally, the infiltration of mast cells is mainly observed at the periphery of nerve endings, which correlated with chronic pelvic pain, suggesting a relation with nociception in this disease that could be produced through the transient receptor potential vanilloid subfamily 1 (TRPV1) channel (82).

The alteration of the mechanism of maturation, infiltration, and functions of immune cells at the systemic or local level in endometriosis is mediated through modification of molecular and the biochemical environment that converges in the progression of the disease. This makes them potential targets to drug design or looking for alternative treatments that inhibit specific molecules of altered pathways involved in exacerbated inflammation and associated symptoms such as pain or infertility.

### Alterations of Immune Mediators

The predominant molecular characteristic of endometriosis is the elevated concentration of immune mediators, mainly pro-inflammatory molecules, both locally and at the systemic level. NF-κB is a key transcriptional factor that is overexpressed and overactivated in endometriotic cells of the lesions and peritoneal macrophages, an effect that is potentiated by the pro-inflammatory environment through action of cytokines. In turn, NF-κB is involved in the positive regulation of these pro-inflammatory factors as well as of chemoattractants, adhesion molecules, and angiogenic factors (83). Immune cells and endometriotic stromal cells are an important source of cytokines, prostaglandins, and chemokines, which are released to the surrounding environment, showing consistency in elevated levels between different fluids and tissues. For example, levels of pro-inflammatory cytokines IL-1β, IL-6, IL-8, IFN-γ, and TNF-α are elevated in peritoneal fluid and serum of patients with endometriosis and could be used as non-invasive markers of the disease (84).

IL-1β is the more studied inflammatory cytokine involved in the pathophysiology of endometriosis, with multiple roles in the disease development. For example, it is involved in activation of cyclooxygenase 2 (COX2), which in turn elevates prostaglandin E_2_ (PGE_2_) levels that contribute to activation of aromatase and other members of steroidogenic pathway, through an increased binding of steroidogenic factor 1 (SF-1) to promoter of aromatase gene (85). IL-1β also contributes to recruitment of macrophages and neuroangiogenesis by regulating chemokine RANTES and promoting the production of brain-derived neurotrophic factor (BDNF), which co-localizes with macrophages and nerve fibers in endometriotic lesions and cultures of eutopic stromal cells; both events are mediated through c-Jun N-terminal kinase (JNK) and NF-κB signaling pathways. These findings support the notion of interaction between pro-inflammatory factors that favor the communication between different cell types in endometriotic lesions, which as a result promote the recruitment of vessels and nerves to stimulate angiogenesis, growth of the lesion, and generation of pain (40).

IL-6 for its part is found at elevated concentrations in peritoneal fluid and circulation; it is secreted by both macrophages and endometriotic cells, with the macrophages being its main source (86, 87). Serum concentration of IL-6 was analyzed along with the concentration of surface antigen CA125, used frequently as a marker of disease, and both showed an association to moderate-severe endometriosis (87). IL-6 also participates in the decrease of differentiation and cytolytic activity of NK cells, by inducing the tyrosine phosphorylation of phospholipase Cg and tyrosine phosphatase SHP-2, whose expression inhibits cytotoxic activities of NKs, promoting survival of endometriotic cells (86). Multiple functions of IL-6 are mediated by its receptor IL-6R, located on macrophages surface, which simultaneously makes them the source and the target of this cytokine (88). Its elevated expression and secretion by peritoneal macrophages from women with endometriosis is promoted by E2, which in turn is associated with induction of growth of endometriotic lesions (55). Additionally, this pro-inflammatory cytokine participates in the stimulation of aromatase expression in stromal cells from lesions (89), which shows again an interaction between the endocrine system and inflammation processes.

In addition to its function as a chemokine, IL-8 has other important roles in endometriosis development. Its elevated concentration has been shown in endometriotic stromal cells, as an effect of the action of E2 and TNF-α through NF-κB; remarkably, this increase is reversed by P4 treatment, indicating that P4 downregulation in endometriosis contributes to alteration of IL-8 expression during the pathogenesis of the disease (90). Moreover, IL-8 considerably increases the proliferation of stromal cells from ovarian endometriomas (91) and shows a positive correlation with the severity of endometriosis.

On the other hand, TNF-α is considered an essential factor in the pathogenesis of endometriosis. It is secreted in high levels by pelvic macrophages in response to E2 treatment (55). TNF-α promotes the production of prostaglandins PGF_2_ and PGE_2_ by epithelial and stromal endometrial cells in endometriotic lesions and stromal cells from eutopic endometrium of women with endometriosis by inducing COX2 overexpression through NF-κB activation (92, 93). TNF-α induces endometriosis-associated inflammation by overregulated secretion of other molecules as IL-6, granulocyte-macrophage–colony-stimulating factor (GM-CSF), and MCP-1 (94).

Another pro-inflammatory cytokine involved in endometriosis is IL-17A that stimulates the production of pro-angiogenic cytokines, such as IL-8 or IL-1β, and was found in significantly higher concentrations in the peritoneal fluid of patients with minimal-to-mild endometriosis than those with moderate-to-severe endometriosis and without the disease. Therefore, IL-17 could play an important role in the initial phases of endometriosis by favoring the angiogenesis in the peritoneal surface, which facilitates the survival, implantation, and proliferation of ectopic endometrial tissue (95). In addition to the role in promoting angiogenesis, IL-17 contributes to maintaining a pro-inflammatory environment in the peritoneal cavity needed for the establishment and preservation of endometriosis lesions, where it is produced at elevated levels. Moreover, studies have shown that IL-17 plasma levels are also increased and are dependent on the stage of the disease. This cytokine also maintains the inflammatory and angiogenic milieu by inducing the production of cytokines VEGF, platelet-derived growth factor (PDGF)-AA, C-X-C motif chemokine ligand 12 (CXCL12), and granulocyte colony-stimulating factor (G-CSF) (96).

IFN-γ is also expressed at elevated levels in ectopic endometrium in contrast to eutopic endometrium of patients with endometriosis; a similar situation is observed in serum as well as in peritoneal fluid of patients (97–99), which is related with an unbalanced immune activity in endometriosis. IFN-γ is involved in increasing the resistance of endometrial cells to apoptosis and to stimulate the expression of cell adhesion molecules to allow the establishment of endometriotic lesions (99).

Prostaglandins are locally produced hormones involved in inflammation and pain. In women with endometriosis, PGE_2_ and PGF_2α_ are produced in elevated concentrations both in the eutopic and ectopic endometrium. PGF_2α_ contributes to dysmenorrhea through its vasoconstrictive properties and the capacity to induce uterine contractions. In contrast, PGE_2_ may induce pain directly. In endometriosis, there is a positive feedback between inflammation mediators and E2. This hormone stimulates COX2 activation that increases PGE_2_ production using arachidonic acid as precursor; PGE_2_ production in turn influences steroidogenic genes, mainly overexpressing aromatase (100), therefore increasing the E2 levels that stimulate signaling through ER-β. As mentioned before, the inflammatory microenvironment in endometriotic lesions also stimulates PGE_2_ production (65). Interestingly, COX2 is differentially regulated in ectopic and eutopic endometrium from endometriosis patients in response to IL-1β, since ectopic tissue is more sensible to this induction that is important to sustain inflammatory microenvironment that in turn sustain lesion development. This increased sensibility is due to IL-1β capacity to increase COX-2 transcript stability (65, 100). Angiogenic factor VEGF also participates in PGE_2_ production by induction of COX2 expression (101), which shows the interplay between inflammation and cell pathways that sustain the maintenance of endometriotic milieu.

Chemoattractants facilitate the recruitment of macrophages at the lesion site, especially chemokines IL-8, MCP-1, and RANTES, whose participation was described in section alteration of immune cells function, which can serve as biomarkers in identifying patients with endometriosis, but the precision of such tests can be enriched by including other inflammatory markers, or in the case of infertility-associated endometriosis, these molecules could be included as endometrial receptivity markers. Interestingly, MCP-1 is overexpressed (mRNA and protein) by action of P4 and E2 only in endometrial endothelial cells from patients with endometriosis, in contrast with cells from healthy women that did not show MCP-1 expression in response to this stimulus. Therefore, hormonal induction of chemokine expression in the endometriotic cells plays an important role in recruiting mononuclear cells, which contribute to inflammation in endometriosis (102).

The pro-inflammatory environment also has the capacity of regulating expression of steroid hormone receptors; Grandi et al. observed in endometrial stromal cells (eutopic tissue) isolated from endometriosis patients that exposition to TNF-α and IL-1β significantly reduces the expression of both PR-A and PR-B (mRNA and protein). This contributes to P4 resistance, by altering the response to hormonal treatments using progestins, which explains the low local response to this therapy and indicates that the origin of inflammation in endometriosis lies not only in the ectopic tissue but also in the cells of eutopic tissue, whose multiple molecular alterations could allow them to survive out the uterus (57).

On the other hand, elevated expression of immunosuppressive cytokines in endometriosis has also been reported, like IL-4 and IL-10, which are involved in the development of disease through stimulating survival, growth, invasion, angiogenesis, and immune escape of endometriotic lesions (103). The elevated concentrations of these cytokines could be due to their insufficient control over pro-inflammatory activities, overcompensating and inhibiting the immune response. IL-4 is found at high levels in endometriotic tissues, where it stimulates the proliferation of endometriotic stromal cells by stimulating the activation of p38MAPK, SAPK/JNK, and p42/44 MAPK signaling (104). IL-10 in serum and peritoneal fluid from patients with endometriosis has shown significantly increased levels in contrast to those from healthy women (105). The role of this cytokine was addressed using a mouse model of surgically induced endometriosis. The blocking activity of IL-10 in mouse decreases the size of lesions, while the administration of this cytokine promoted their growth; additionally, infiltrated dendritic cells were the main secreting source of IL-10. The foregoing suggests that IL-10 contributes to immune suppression necessary for the development of endometriosis (105, 106).

For its part, retinol, which is the precursor of retinoic acid (RA) necessary for endometrial cell differentiation and function, and whose nuclear receptors have also been detected in endometrium, also has a participation in endometriosis, since genes that regulate its synthesis and signaling pathways have an altered expression, which therefore causes reduction of RA. Cell pathways altered in endometriosis and regulated by local retinoic acid production involve MMP secretion, gap junctional intracellular communication, and the expression of a variety of cytokines involved in cell differentiation and immune regulation. Some altered RA-regulated molecules are IL-6, MCP-1, TNF-α, VEGF, connexin 43, various integrins, and *fas* ligand; this dysregulation is related to the progesterone-resistance effect (107, 108). This observation indicates a relationship between diet and nutrition in the disease, supporting the hypothesis of its multifactorial origin.

The use of biochemical markers of inflammation has been proposed to evaluate disease in complement instead of detection of endometriotic lesions through laparoscopic surgery. In this respect, a nested case–control study revealed that plasma IL-1β levels were positively associated with an increased risk to develop endometriosis. However, an association between plasmatic IL-6 and TNF-α levels with disease risk was not found (109), but an association of TNF-α with this risk was found only between women younger than 40, suggesting that young age is a threat factor to develop endometriosis, and the authors concluded that IL-1β and TNF-β could be early markers of disease. Recently, significantly higher concentrations of SCGF-β, IL-8, HGF, and MCP-1 have been reported, as well as lower levels of anti-inflammatory cytokine IL-13 in endometriosis patients such as those found in women without endometriosis (110). Additionally, in the same study, through regression analysis, the combination of SCGF-β, IL-13, and G-CSF concentrations predicted with accuracy the presence of endometriosis (86% sensitivity and 67% specificity) (110). Other studies focused to associate concentrations of pro-inflammatory cytokines and markers of receptivity in peritoneal fluid, such as IL-8, RANTES, osteoprotegerin (OPG), pregnancy-associated plasma protein-A (PAPP-A), TNF-α, midkine, and glycodelin, with the severity of pain according to pain scores. Concentrations of these molecules were found to be correlated with pain level and stage of endometriosis; in particular, this correlation was positive in the case of increased levels of TNF-α and glycodelin, indicating the potential role of this molecule in the severity of pain in endometriosis (111).

In summary, dysregulation of immune molecule expression is manifested both in immune system cells and in endometriosic cells, in response to an altered hormonal environment, which in turn responds to changes in the immune milieu. In fact, the action of these molecules is not individual; they act in a coordinated fashion to regulate the production and function of each other, thus forming a complex network that allows the communication between different cell types in order to maintain the viability and development of endometriotic lesions. Due to the importance of these inflammatory factors, it is necessary to perform more studies about altered immune factors associated with the severity of endometriosis. Since most molecules found in ectopic tissue can also be found at systemic level, that is, they could reflect the local environment that surrounds endometriotic lesion, it is fundamental to propose a complete panel of non-invasive biomarkers to detect early stages of the disease in patients who are exposed to commonly associated risk factors or have a family history of endometriosis, which could be useful to complement or substitute the surgical diagnosis. In the same way, this knowledge will be advantageous to determine the appropriate therapeutic target and treatment to each patient, in the process of designing a personalized therapy.

## Effects of Sex Hormones on Inflammasome Regulation in Endometriosis

Inflammasomes are complex molecular structures from the innate immune system that control pyroptosis (inflammatory form of cell death) and the activation of caspase 1, which is an enzyme involved in the proteolytic maturation of the pro-inflammatory cytokines IL-1β, IL-18, and IL-33, which, by caspase-1 influence, are released in a non-classical secretion pathway (112). Production of IL-1β and IL-18 occurs in response against components of pathogen microorganisms (pathogen-associated molecular patterns, PAMPs) or danger signals from damaged and necrotic cells (danger-associated molecular patterns, DAMPs) that activate pattern recognition receptors (PRRs) expressed on the surfaces of macrophages, dendritic, and epithelial cells, among which are Toll-like receptors (TLRs) (113). After recognition of PAMPs or DAMPs by PRRs, adapter molecules are recruited, leading to NF-κB activation and, in consequence, the overexpression of genes that encode pro-inflammatory cytokines. For activation of caspase 1, the assembly of a complex integrated by a cytosolic sensor is necessary [a PRR that consists in a nucleotide-binding domain and leucine-rich-repeat-containing (NLR, also known as NOD-like receptor) or an AIM2-like receptor (ALR) protein], an adapter protein known as apoptosis-associated speck-like protein containing a CARD (ASC), which interacts with procaspase 1, connecting it with sensor that initiates self-cleavage of caspase 1 and the formation of its active form that is necessary for processing of immature cytokines (114).

Nevertheless, alteration of inflammasome is involved in the development of distinct diseases related to inflammatory disorders, for example, inflammatory bowel disease, Crohn's disease, vitiligo, periodontitis neurodegenerative diseases such as multiple sclerosis, Alzheimer's disease, and Parkinson's disease, as well as metabolic disorders that include atherosclerosis, insulin resistance, type 2 diabetes, and obesity (115). Besides the former illnesses, inflammasome also plays an important role in reproduction pathologies.

Recently, it has been demonstrated that human endometrium expresses inflammasome components, including the NLR family member NLRP3 (also identified as NALP-3) and ASC protein, which are significantly overexpressed in the endometrium of patients with recurrent pregnancy loss (without any evidence of infection) in contrast to healthy fertile women. In consequence, caspase 1 showed an increased activity and augmented levels of both IL-1β and IL-18 were detected, which probably led to an abnormal activation of uterine innate immunity that is involved in the establishment of a pro-inflammatory endometrial milieu, which would impede successful implantation, and provoke disturbance of placental development and pregnancy loss (116).

Inflammasome pathways could also be involved in the pathophysiology of endometriosis. Recently, to investigate the role of non-genomic activation of ER-β in this disease, a mouse model of surgically induced endometriosis was used through autotransplantation of fragments of endometrial tissue in the peritoneal cavity, both in mice with overexpression of ER-β (ERβ-OE) and in ERβ-null mice. Lesions in ERβ-OE mice had greater dimensions than in ERβ-null and control mice, confirmed by using a selective antagonist, suggesting an important participation of receptor in the progression of the disease (31).

In the same study, the authors determined interactions of ER-β with proteins involved in apoptosis, such as apoptosis signal-regulating kinase-1 (ASK-1), which participates in apoptosis induced by TNF-α. Nonetheless, ASK-1 has shown reduced activity in ERβ-OE mice; additionally, an ASK-1 interaction with serine/threonine kinase receptor-associated protein (STRAP) and 14-3-3 protein was observed, a complex that also interacts with ER-β; in turn, the STRAP/14-3-3 complex obstructs ASK-1/TNF receptor-associated factor 2 interaction, hence inhibiting TNF-α-mediated apoptosis.

In the same way, the interaction of ER-β with inflammasome components was determined, such as NLRP3 and caspase 1. The processed form (and therefore its activated form) of the latter along with IL-1β was found in high levels in endometriosis lesions of ERβ-OE mice. This is in accordance with previous reports of endometriosis in humans, in which it has been observed that endometriotic implants produce elevated concentrations of IL-1β, in contrast to eutopic endometrium or endometrium of healthy women; likewise, this increase is also observed in the peritoneal fluid of endometriosis patients (117, 118). In conclusion, the mechanism observed in this model of endometriosis progression indicates an interaction between the immune system and estrogen signaling through non-genomic ER-β activity. This interaction, in which ER-β cooperates with components of inflammasome machinery to increase IL-1β levels and to modulate apoptosis induced by TNF-α, activates a mechanism to ensure endometriotic cell survival, adhesion, and proliferation through evasion of immune surveillance and the epithelial–mesenchymal transition pathway (31). However, the interplay between non-genomic and genomic activities of ER-β, as well the participation of ER-α and other steroid receptors in inflammatory processes of endometriosis, remains to be determined. In addition, key components of inflammasome and their interactions with these receptors could be useful as drug targets, designed to interrupt downstream signaling pathways, thus affecting progress of the disease and maintaining it in stages easily controlled by using non-invasive therapies.

## Contribution of Inflammation Of Bacterial Origin to Endometriosis

Endometriosis is a multifactorial disease in whose development alteration of sex hormone pathways has a key role and bacterial infections of the female reproductive tract or alterations in its microbiota composition seem to have significant participation. Inflammation in endometriosis has been related to the presence of bacteria and their products (metabolites or virulence factors) in the peritoneal cavity, which led researchers to propose the “bacterial contamination” hypothesis (119). Some observations indicate the presence of *Escherichia coli* in menstrual blood of patients diagnosed with endometriosis in contrast with control women; bacterial load correlates with elevated concentrations of LPS in menstrual blood and peritoneal fluid. In addition, the concentrations of HGF, VEGF, IL-6, and TNF-α were significantly higher in the culture media of peritoneal macrophages treated with LPS from *E. coli*; this effect was mediated through activation of TLR4; this receptor was also detected in stromal and gland cells from eutopic or ectopic endometrium from endometriosis patients (120). The authors suggested that resident Gram-negative bacteria of the vagina, including *E. coli*, could migrate to the upper reproductive tract and have an involvement in endometriosis by the accumulation of endotoxin in the menstrual/peritoneal fluid that would unchain pelvic inflammation, leading to growth of lesions. High concentrations of PGE_2_ in peritoneal or menstrual fluids could be a factor that promotes bacterial survival since it stimulates bacterial growth *in vitro* and simultaneously causes impairment of the function of lymphocytes (121).

Taking these results as antecedent, using a mouse model of endometriosis, it was demonstrated that simultaneous administration of blood (simulating menstrual reflux that reaches pelvic cavity during retrograde menstruation) with LPS in mice, which were previously injected with endometrial implants, induces the growth of endometriotic lesions and the production of TNF-α, IL-6, and MIP-2 in peritoneal fluid (122), which in turn is related with NF-κB expression (123). An additive effect has been observed with the simultaneous treatment of macrophages with LPS and E2, which induced secretion of pro-inflammatory factors and proliferation of endometriotic cells (55). The above suggests a role of LPS in coordination with steroid hormones in the first stages of endometriosis development through induction of inflammatory responses and recruitment of neutrophils to endometriotic lesions, which in turn possibly participate in the release of angiogenic factors, unchaining a process of inflammatory angiogenesis (122). These observations have to be taken carefully since findings can be caused by an asymptomatic or subclinical vaginal infection or contamination with bacteria from cervicovaginal microbiota that contribute to bacterial contamination of analyzed samples (124).

On the other hand, the role of the microbiome in the pathogenesis of endometriosis has also been suggested; however, the role of this microbial community in endometrial function and the development of its pathologies remain unclear. In contrast to the general acknowledgment about uterine cavity as a sterile environment, recently by the usage of next-generation sequence tools in combination with culture techniques, it has been reported that endometrium has its microbiota (125); however, it is still debated whether the bacterial presence is due to contamination from the vaginal or cervical microbiome (126).

Most of the studies have reported the presence of members of phyla Firmicutes, Bacteroidetes, Proteobacteria, and Actinobacteria, among others, as part of “health” uterine microbiota (127–129), and that changes of this diversity and its abundance are related to alterations in endometrial function (127, 130, 131), benign uterine pathologies (128, 132, 133), or endometrial cancer. In the case of endometriosis, changes of taxonomic bacterial composition to potentially pathogen bacteria in patients diagnosed with the disease have been detected in peritoneal fluid, cervical mucus, and cervicovaginal microbiota (129, 134, 135). However, studies that detect the real changes in endometrial microbiota related to endometriosis are lacking; furthermore, studies carried out to date in general lack a control group with healthy women (without any benign pathology) and face difficulties to obtain endometrial samples, among other technical complications (126, 134, 136).

On the other hand, although the mechanisms used by microbiota of the upper reproductive tract to regulate host functions are still unidentified, it has been suggested that there is a relation between uterine microorganisms and the uterine immune system, and it is speculated that bacteria interact with endometrium and control the expression of endometrial factors involved in inflammatory response, proliferation, apoptosis, expression of leukocyte subsets, infiltration of plasma cells, and secretion of immunoglobulins, which in turn regulates processes of decidualization, embryo implantation, pregnancy development, and protection against infections (116). More studies are necessary to confirm and characterize the composition and the role of microbiota on the physiology of the human female reproductive system, particularly in the endometrium, and its relationship with components of uterine microenvironment, as hormonal levels and metabolites are produced at the endometrium. In the future, this knowledge could enable the potential use of bacterial microbiota as a diagnostic tool and as a therapeutic target.

## Association of Inflammation with Infertility in Patients with Endometriosis

Chronic inflammation is strongly linked to alterations in female fertility, as occurs with infertility-associated endometriosis, where the produced “hostile” environment alters the sperm transport, tubal motility, and oocyte development (through impaired uterine peristalsis), as well as embryo implantation (137). At the uterine level, inflammation induces an impairment of endometrial decidualization as a response to progesterone resistance due to the alteration in the function of PR. Therefore, the expression of progesterone-responsive genes is dysregulated during the implantation window, which comprises a period of 8–10 days after the luteinizing hormone (LH) surge (mid-secretory phase) (11). Some of these altered genes encode biomarkers of endometrial receptivity (138), which are involved in embryo attachment and stimulation of decidua invasion, such as adhesion molecules (CAM family), cytokines, growth factors, and prostaglandins (11).

Decidualization occurs during the secretory phase of the menstrual cycle in response to ovarian hormones, conducive to differentiation and specialization of the endometrium, independent of the presence of a fertilized oocyte, initiating a series of profound changes that allow embryo implantation and is finished when the development of the placenta is complete (139). At the molecular level, changes reflected in gene expression, proteome, and secretome occur. In particular, two robustly secreted molecules are the hallmark of decidualization: insulin-like growth factor binding protein-1 (IGFBP-1) and prolactin (PRL) (140), whose expression is related to PR activity (141, 142). Both IGFBP-1 and PRL are drastically downregulated in the eutopic endometrium of women affected by endometriosis (143). In addition, IGFBP-1 and PRL also show a lower secretion by cultured endometrial stromal cells from women with endometriosis than those from healthy women (144).

CAM encompasses integrins, cadherins, selectins, and immunoglobulins, which typically are glycoproteins involved in cell-to-cell adhesion; during the implantation window, they lead to firm attachment of the blastocyst to the endometrial pinopods to guarantee successful implantation (138). Between CAMs is α*νβ*3 integrin, a molecule that suffers dynamic alterations over the course of the menstrual cycle, with a peak of expression at the mid-secretory phase. α*νβ*3 is activated upon binding to its ligand osteopontin, and forms aggregates that recruit cytoskeletal proteins and components of cell signaling pathways required for attachment of embryo. Endometrial α*νβ*3 integrin expression was significantly reduced in patients with endometriosis during the window of implantation (145), probably in response to local estrogen (146). The diminished αvβ3 expression is related to impaired production of the HOXA10 transcription factor (member of family homeobox, which is involved in the development and physiology of the uterus) that regulates expression of subunit β3 (147, 148). Reduced expression of HOXA10 is related to epigenetic changes, and there exists evidence of hypermethylation of its promoter and its consequent silencing in the eutopic endometrium of endometriosis patients, in comparison to healthy endometrium (149). Also, HOXA10 regulates IGFBP-1 expression, making it a key transcription factor during the process of uterine receptivity maintaining (150).

HOXA11 is another member of the homeobox family that is involved in diverse processes associated with embryo development and aspects of female reproductive tract physiology such as endometrial growth, differentiation, receptivity, and embryonic development. HOXA11 is detected at significantly lower levels (mRNA and protein) in the eutopic mid-secretory endometrium of infertile patients with endometriosis than in endometrium of fertile women. HOXA11 promoter is hypermethylated; this can be one of the possible molecular mechanisms causing a decrease in its expression. Obtained results indicate the relation of HOXA11 with infertility in endometriosis (151). A previous report supports this observation since HOXA11 seems to regulate PRL expression during decidualization (152). It is to be noted that regulation of expression of HOXA genes in healthy endometrium is mediated by sexual hormone action, mainly P4, showing a significant increase at the secretory menstrual phase (153, 154), which suggests that its dysregulation in endometriosis is also related to alteration in hormonal pathways.

Further transcriptional regulators involved in uterine receptivity are down-regulated in eutopic endometrial tissue from endometriosis patients. These include GATA Binding Protein 2 (GATA2), SRY-Box 17 (SOX17), Indian hedgehog signaling molecule (IHH), COUP transcription factor 2 (COUPTFII), Wingless-type MMTV integration site family (WNT4), Forkhead box O1 (FOXO1), AT-rich interaction domain 1A (ARID1A), and histone deacetylase 3 (HDAC3), most of which are involved in regulation of P4-responsive pathways (17).

Among other alterations observed in CAMs of eutopic endometrium, there is an altered regulation of the E-cadherin and β-catenin expression in epithelial cells of endometrium from infertile women with endometriosis during the mid-secretory phase, since both are expressed at increased levels (155). The β-catenin expression is associated with the Wnt pathway, which could be activated in endometriosis. In the fertile endometrium, increased E2 levels induce Wnt/β-catenin signaling to enhance proliferation, whereas P4 inhibits this signaling pathway during the secretory phase, reducing proliferation and promoting cell differentiation (156); however, in endometriosis, it is probable that P4 resistance and hyper-estrogenic milieu prevent inactivation of this pathway, thus promoting cell proliferation while inhibiting decidualization (155).

Glycodelin A, an immunomodulator glycoprotein regulated by P4 during the window of implantation, is downregulated in endometrium of women with endometriosis (143, 157). However, there are inconsistencies between published reports; for example, an analysis of expression reveals an increase in glycodelin A levels in the eutopic endometrium of patients with endometriosis in contrast to those from the endometrium of control group (158). This coincides with its elevated concentrations found in peritoneal fluid and serum of endometriosis patients compared to controls, in both proliferative and secretory cycle phases; importantly, values of concentration correlate with level of pain intensity (159). Differences could be attributed to the type of endometriotic lesion and the severity of the disease or ethnicity of the study population, even though general evidence suggests that glycodelin A has a significant participation in uterine receptivity and could be useful as a non-invasive biomarker of endometriosis.

Among altered cytokines, leukemia inhibitory factor (LIF), a member of IL-6-like family E2-responsive, essential for blastocyst implantation, also shows reduced expression in infertile patients with endometriosis, especially at moderate stages of the disease (160, 161). Another IL-6-like family member required for embryo implantation, IL-11, and its receptor, are absent in the glandular epithelium of endometrium from endometriosis patients, in contrast to fertile women (161). Another cytokine involved in endometrial infertility is IL-1β, which can alter the differentiation of stromal endometrial cell by causing disruption of decidual function through cellular depletion of ER-α, PR, and gap junction alpha-1 protein (also known of connexin 43 or Cx43); inhibition of IL-6-mediated extracellular signal-regulated kinase (ERK)1/2 pathway recover decidualization markers as well as production of steroid receptors (162).

Among other receptivity markers altered in endometriosis are the transcriptional gene repressor BLC6 and the histone deacetylase sirtuin 1 (SIRT1), whose co-expression is promoted by pro-inflammatory environment in eutopic endometrium from endometriosis patients; both are used for the diagnosis of disease. BLC6 and SIRT1 overexpression (induced by Kirsten rat sarcoma viral oncogene, KRAS) alter the actions of P4 by suppression of the promoter of *GLI1*, a critical mediator of progesterone action in the Indian Hedgehog pathway, contributing to the pathophysiology of this disease and infertility through the increase of P4 resistance (163).

Overall, receptivity endometrial markers show a strong interaction with components of pro-inflammatory and hormonal environment, which influence negatively their expression and function and hence contribute to impede a successful pregnancy in endometriosis patients. In order to preserve women's fertility and to promote effectiveness of *in vitro* reproduction techniques, it is necessary to restore the adequate expression and production of molecules involved in uterine receptivity, through safe and personalized immunomodulatory therapies that reduce or avoid surgical interventions and their adverse effects. Moreover, it is necessary to establish an integral management of the disease according to individual requirements of each patient, which encompasses non-invasive diagnosis and medical and psychological therapies, through the interactions of researchers in basic molecular biology with distinct clinical specialists.

## Current and Future Treatments for Inflammation Associated with Endometriosis

The treatment more extensively used for endometriosis is hormone modulation by progestins that can bind PR and regulate its activity, thus suppressing ovulation, provoking amenorrhea, and a hypo-estrogenic environment; however, not all the patients that receive the treatment have benefited from it. Another common treatment is the inhibition of production of prostaglandins, through a COX or an aromatase inhibitor, for example, a non-selective and non-steroidal anti-inflammatory drug such as ibuprofen or naproxen, which significantly reduce symptoms of disease, mainly pelvic pain. However, they can produce secondary effects such as the development of osteopenia, osteoporosis and bone fractures, increased cardiovascular risk, and negative gastrointestinal effects (6, 53). To ensure the efficiency of treatment and avoid undesirable secondary effects, the compound used should be effective on specific targets of distinct affected pathways simultaneously and ideally originated from natural sources. In this section, we describe selected potential drugs and alternative treatments with these characteristics, which are recently reported in the literature.

Resveratrol, a polyphenolic compound with anti-proliferative and anti-inflammatory actions, found in many dietary sources, is an alternative for endometriosis treatment (164). In a rat model with experimentally induced endometrial implant, resveratrol significantly reduced implant areas, while it decreased serum and peritoneal levels of VEGF and MCP-1. For this reason, resveratrol is considered a potential novel therapeutic agent that acts by inhibition of angiogenesis and inflammation (165).

Crocin is a vegetal compound found in some flowers and saffron, and it possesses anti-inflammatory and anti-proliferation properties. In a mice model of endometriosis with crocin treatment, aspects of inflammation, angiogenesis, growth of lesions, as well as endothelial apoptosis and proliferation were evaluated. Crocin avoids the growth of the lesion through inhibition of proliferation without causing apoptosis, as well as reducing the expression and secretion of inflammatory cytokines INF-γ, TNF-α, and IL-6, as well as VEGF and proliferating cell nuclear antigen (PCNA) (166).

Another natural compound is nobiletin, a flavonoid isolated from citrus peels with capacity for inhibiting NF-κB activation. A recent study in mice shows that administration of nobiletin significantly reduced lesion size and expression of PCNA, VEGF, E-cadherin, IL-6, IL-1β, TNF-α, MMP-1, and MMP-3; this observation was related with the inactivation of NF-κB through targeting the activity of IκB kinases (167).

Recently, molecules isolated from sources used in traditional medicine have also been reported; this is the case of dehydrocostus lactone from the roots of *Aucklandia lappa*, used in Asiatic traditional medicine for the treatment of diseases associated to inflammation and pain. This compound inhibited Akt and NF-κB pathways in endometriotic cells and macrophages. Dehydrocotus lactone caused apoptosis in endometriotic cells by activation of caspase-3, -8, and -9, and decreased the production of PGE_2_ and neurotrophins, while that in macrophages induced the decrease of IL-10, VEGF, MMP-2, and MMP-9 (168).

On the other hand, therapies can be implemented by using existent drugs used to treat other inflammatory diseases. Simvastatin, which belongs to the statins family, was assayed in a baboon model of endometriosis. Treatment with simvastatin induced ER-α expression while provoking a reduction of ER-β in lesions and eutopic tissue. Furthermore, simvastatin significantly reduced the expression of neopterin, a marker of inflammation, oxidative stress, and immune system activation. Collectively, the present findings indicate that the inhibition of the mevalonate pathway by simvastatin reduces the risk of developing endometriosis in the primate model of this disease by decreasing the growth of endometrial lesions, by modulating the expression of genes encoding for estrogen receptors, and by reducing inflammation (169).

Regardless of the finding of new potential therapies for endometriosis treatment, it is necessary to perform more studies to determine the effectiveness of therapeutical agents in women and to assess possible side effects on reproductive tract function. Additionally, these discoveries can enhance the activity of traditional drugs if used simultaneously with them.

## Conclusion

Endometriosis is a complex gynecological disease characterized by a chronic inflammatory process that is strongly linked to altered sex hormonal-dependent pathways, mainly through steroid hormone receptor function (Figure 1). According to current evidence, its origin is multifactorial, involving molecular, biochemical, and cellular alterations that in turn are interconnected and possibly related to responses to the external stimuli; however, it is not yet determined whether these alterations are the consequence or the effect of the disease. It is necessary to elucidate the key components of each of the involved pathway in the development of endometriosis, particularly those related to inflammation, responsible for the activation of other important cellular pathways involved in the development and maintaining of the endometriotic lesions, which in turn contribute to the maintenance of the inflammatory milieu. Further studies are still necessary for elucidating the pathogenesis of the disease, by translating animal and *in vitro* models to clinical studies that mimic the disease in humans the nearest. This knowledge will allow to determine the ideal targets for developing novel therapies to treat endometriosis effectively, through recovery of altered cell functions and that at the same time avoid recurrence of the implants or undesirable secondary effects. In turn, these targets ideally must be accurate non-invasive biomarkers for the diagnosis and classification of the disease. For this end, it is imperative to develop biomarker panels that ideally must contain individual molecules involved in interconnected pathways, as well as molecular complexes or bacterial signatures, which in turn will contribute to the selection of personalized treatments according to disease particularities and/or fertility desire in each patient, looking to improve her life quality.

## Author Contributions

EG-G, EV-M, and MC designed the concept. EG-G and CR-M wrote the first draft of the manuscript. IC-A and OC-O revised and corrected the first version of the text. All authors read, revised, and approved the manuscript for publication.

### Conflict of Interest

The authors declare that the research was conducted in the absence of any commercial or financial relationships that could be construed as a potential conflict of interest.
